# Fixed-time artificial insemination protocols on brazilian locally adapted breed gilts on ovulatory response and embryo production

**DOI:** 10.1590/1984-3143-AR2020-0776

**Published:** 2021-05-28

**Authors:** Priscilla Cristine Passoni Silva, Oscar Oliveira Brasil, Paula Lorena Grangeira Souto, Nathalia Hack Moreira, Joseane Padilha da Silva, Bianca Damiani Marques Silva, Alexandre Floriani Ramos

**Affiliations:** 1 Departamento de Ciências Animais, Faculdade de Agronomia e Medicina Veterinária, Universidade de Brasília, Brasília, DF, Brasil; 2 Embrapa Recursos Genéticos e Biotecnologia, Brasília, DF, Brasil

**Keywords:** synchronization protocol, fixed time artificial insemination, embryo recovery, pig, genetic resources

## Abstract

The aim of this study was to use estrus synchronization protocols to favor fixed-time artificial insemination and consequently fixed-time embryo collection, and increase embryo production using eCG, in gits. In a cross over design, nine *Piau* breed gilts were subjected to 18 days of oral progesterone; P4 group did not receive any further; GnRH group received 25µg of GnRH 104 hours after the final application of P4; and eCG+GnRH group received 1000IU of eCG 24 hours after the final P4 in addition to GnRH for subsequent embryo collection, that was performed six days after first AI, by laparotomy. Artificial insemination was performed after 12 and 24 hours of estrus in P4 group, and 128 and 144 hours in GnRH and eCG+GnRH groups. The number of CL (8.6±3.9; 8.3±2.1; 26.7±15.0) and anovulatory follicles (4.3±3.7; 3.9±3.9; 17.2±9.5) was higher in the eCG+GnRH gilts (*P*<0.05). However, the use of 1000 IU of eCG reduced (*P*<0.05) the number of total structures (5.2±3.6; 5.1±3.1; 1.7±2.7), viable embryos (5.0±3.5; 4.8±3.3; 0.4±0.7), freezable embryos (3.6±3.4; 3.3±3.8; 0.1±0.3) and recovery rate (63.7±38.9; 58.6±24.7; 5.38±9.5). P4 and GnRH protocols were effective in the production and recovery of embryos. However, the use of 1000 IU of eCG, 24 hours after P4, was not effective in promoting the production of embryos, although the animals had superovulated.

## Introduction

At the beginning of the 21st century, there is a change in the demand of the consumer market, mainly for the production of human or animal food, where there is an increase in the demand for food produced with little interference in the natural growth and termination process, making it possible to maintain the animal welfare, reducing breeding stress and indiscriminate use of medicines. Given this, there is an opportunity for the genetic material of locally adapted breeds to be used in production to obtain more rustic animals and adaptable to the outdoor production system. Besides being less demanding in diet, being easier to handle, and having unique organoleptic characteristics in their meat and bacon production, have a great deal to contribute to pig farming, promoting balance between productivity and adaptability (Mariante and Egito, 2002).

One way to reproductive promote these animals, once the Piau breed has low prolificity, is via the collection of embryos to ensure the genetic variability and prevent the extinction of those breeds (Mariante and Egito, 2002). Some problems encountered in the reproductive management in cyclic gilts is the great temporal dispersion of the onset of estrus, estrus detection and time of artificial insemination. Consequently, synchronization protocols have been developed to concentrate the estrus in swine, contributing to reproductive performance of gilts and sows, since homogeneity in the onset of estrus results in the standardization of the time of insemination and collection of embryos in addition to the increase in the number of viable embryos collected (Tummaruk et al., 2011; Kraeling and Webel, 2015).

The available hormones frequently used to synchronize estrus in swine are the orally progestin, used to synchronize estrous cycle, GnRH to synchronize ovulation, eCG for superovulation (Martinez et al., 2019; Peltoniemi et al., 2019) and a combination of eCG with hCG, primarily to synchronize swine which are weaning and stimulate estrus in gilts (De Rensis et al., 2003). However, there are few studies in gilts and these studies have inconsistency results in FTAI protocols.

Other studies were carried out on Piau breed, but none evaluate FTAI protocols and their effects on embryo production in a fixed time embryo collection. Considering the importance of ensuring genetic variability of local breeds a protocol that more accurately synchronizes estrus and ovulation, allowing a fixed time artificial insemination, is needed to optimize, facilitating work and enabling an increase in embryo production and embryo collection in a fixed time as well. This study had two aims, 1- use estrus synchronization protocols to allow fixed-time artificial insemination and consequently fixed-time embryo collection, 2- associating use of eCG to increase embryo production in less prolific breeds such as locally adapted Piau.

## Materials and methods

### Animals and estrus synchronization

The experiment employed a cross over design, such that each animal received three different treatments during three different time periods. A rest period of 60 days was provided between the end of one treatment and the beginning of the next. Nine gilts of the Piau breed, clinically healthy, cyclical, between 12-24 months old, weighing between 60-110 kg, underwent a three protocols of estrus synchronization for subsequent collection of embryos: Group 1 (P4 Group) – oral administration of 20 mg of progestin (Altrenogest, Regumate®, Intervet Schering - Plough Animal Health), for 18 days; group 2 (GnRH Group) – oral administration of 20 mg of progestin (Altrenogest) for 18 days in addition to a single dose 25 µg GnRH (Gestran®, União Química Farmacêutica Nacional S.A) IM 104 hours after the last dose of P4 and group 3 (eCG+GnRH Group) – oral administration of 20 mg of progestin (Altrenogest) for 18 days in addition to single dose IM of 1000 IU of eCG (Novormon®, Intervet Schering-Plough do Brasil S.A.) and 25µg of GnRH, 24 hours and 104 hours after the final dose of progestin, respectively.

### Estrus detection and fixed-time artificial insemination

Estrus detection was performed in all gilts initiated 24 hours after P4 cessation, performed by experienced personal, twice a day, allowing contact of females with three mature boars for 30 minutes.

The animals of the P4 group were inseminated 12 hours after the onset of estrus and the insemination was repeated 24 hours later. The animals of the GnRH and eCG+GnRH groups were inseminated at a fixed time 128 hours and 144 hours after the last dose of progestin. The animals were inseminated using a pool of refrigerated semen from three boars where each boar contributed the same amount of semen to the formation of the pool. The semen was collected on the day of the first insemination of each gilt using the penile massage technique, and diluted in Beltsville Thawing Solution (BTS), so that each insemination dose contained 100 mL and a 3 billion spermatozoa concentration with at least 70% of total motility and 70% of spermatozoa with normal morphology. Semen was cooled to 15°C.

### Embryo collection

Six days after the first artificial insemination the animals were taken to the operating room for embryo collection. The animals were tranquilized with azaperone (2mg/kg/IM) and anaesthetized with ketamine (15mg/kg/IV) and placed on a surgical table in a *Trendelenburg* position. The uterine horns were exposed by means of a mid-ventral incision and the ovaries were examined to measure ovulation. A 14G Foley catheter connected to a Petri-dish was inserted 25 cm from the utero-tubal junction (UTJ). A 16G intravenous catheter was inserted next to the UTJ, through which 60 mL of PBS at 37°C were introduced. The uterine horns were massaged to assist in recovering the embryos. After total recovery of the PBS, the procedure was repeated for the other horn. After embryo collection, the gilts received a single dose IM of 150 µg of cloprostenol sodium (Sincrocio®, Ourofino).

Animals with ≥ 10 ovulations were considered superovulated. Collected structures were classified according to the stage of development and quality following the morphological parameters established by the International Embryo Transfer Society (Stringfellow and Seidel, 1998). The fertilized structures were classified as viable embryos, the embryos classified as freezable were morula or early blastocysts with quality grade between I and II.

### Statistical analysis

The variables were tested for normality using the Shapiro-Wilk test and for homoscedasticity using the Bartlett test. All of the variables evaluated did not show normal distribution and/or homoscedasticity and were analyzed using the nonparametric Kruskal-Wallis test and the averages thereof were subjected to the t-Student test. The variables of the spread for the onset of estrus were analyzed using the F test. Differences were considered significant when P < 0.05.

This experiment was approved by the Committee on Ethics in the Use of Animals of the Brazilian Agricultural Research Corporation (Embrapa) Genetic Resources and Biotechnology Department (Protocol 007/2014).

## Results

**Figure 1:**
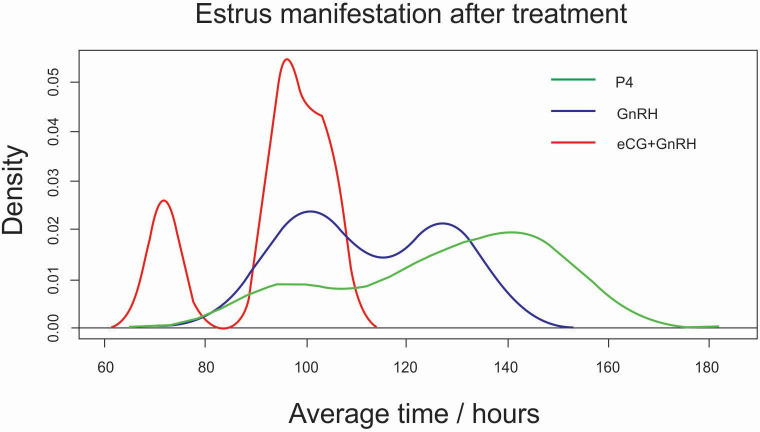
Distribution of the onset of estrus in gilts subjected to the protocols containing P4, eCG and GnRH for subsequent collection of embryos, the x-axis indicates the average duration of estrus (hours) after withdrawal of P4 (*P*> 0.05).

All the animals that showed clinical signs of estrus within 168 hours after the last dosage of P4 were considered synchronized. The animals manifested estrus behavior in the period between 96 and 152 hours, 96 and 128 hours, and 72 and 104 hours, for the P4, GnRH, and eCG+GnRH groups, respectively. Average time for the onset of estrus was 128 ± 20.78 hours in P4 group, 112.88 ± 14.66 in GnRH group, and 93.33 ± 12.64 in eCG+GnRH group. The distribution in the onset of estrus was similar between treatments (*P*>0.05) (Figure 1).

The synchronization protocols significantly influenced (*P* <0.05) the amount of corpus luteum (CL), number of anovulatory follicles (> 8 mm) (Anov. Foll.), total structures, viable embryos, freezable embryos and embryo recovery rate (Table 1). The animals which were stimulated using eCG (eCG+GnRH group) showed an increase in the number of anovulatory follicles and CL, but a decrease in embryo production.

**Table 1:** Mean values and standard error (X ± SE) of ovulatory response and embryo recovery in gilts subjected to protocols containing P4, eCG and GnRH.

**Group**	**CL**	**Anov. Foll.**	**Total Structures**	**Viable Embryos**	**Freezeable Embryos**	**Recovery Rate (%)**
**P4** (n=9)	8.6 ± 1.3^b^	4.3 ± 1.2^b^	5.2 ± 1.2^a^	5.0 ± 1.2^a^	3.6 ± 1.1^a^	63.7 ± 13.0^a^
**GnRH** (n=9)	8.3 ± 0.7^b^	3.9 ± 1.3^b^	5.1 ± 1.0^a^	4.8 ± 1.1^a^	3.3 ± 1.3^a^	58.6 ± 8.2^a^
**eCG+GnRH** (n=9)	26.7 ± 5.0^a^	17.2 ± 3.2^a^	1.7 ± 0.9^b^	0.4 ± 0.2^b^	0.1 ± 0.1^b^	5.4 ± 3.2^b^

^a,b^Different letters in the same column differ between treatments (*P*<0.05). Anov. Foll.= Anovulatory follicles; CL = corpus luteum; P4 group: oral administration of 20 mg of progestin for 18 days; GnRH Group: oral administration of 20 mg of progestin or 18 days in addition to 25 µg GnRH 104 hours after the last dose of P4; eCG + GnRH Group: oral administration of 20 mg of progestin for 18 days in addition to 1000 IU of eCG 24 hours after the final dose of P4 and 25µg of GnRH 104 hours after the final dose of progestin.

## Discussion

The onset of estrus is an effective tool to evaluate the responsiveness of the animals to synchronization treatments. In this experiment, 100% of the animals manifested estrus within the predetermined period for all three treatments, demonstrating that all protocols were effective in synchronizing estrus, which agrees with the results found by other authors (Martinat-Botté et al., 1995, 2010; Soede et al., 2011; Grégoire et al., 2012; Kirkwood and Kauffold, 2015; De Rensis and Kirkwood, 2016). Given that the animals tended to manifest estrus up to seven days after the cessation of oral progesterone and the consequent physiological increase in oestradiol, the onset of estrus in this study and in others is probably influenced by follicular condition at the time of progestagen removal than follicular stimulation with eCG or ovulation induction with GnRH (Martinat-Botté et al., 1990, 1995; Koutsotheodoros et al., 1998; Grégoire et al., 2012; Kirkwood and Kauffold, 2015; De Rensis and Kirkwood, 2016).

As the estrus synchronization protocols were effective in inducing estrus, it was possible to perform artificial insemination at a fixed time, consequently the embryo collections occurred at predetermined times and there was recovery of viable embryos. Embryo production in P4 and GnRH groups was compatible with the prolificacy of the breed (Silva et al., 2014; Montes et al., 2018) and therefore can be considered a satisfactory result since the use of protocols permits better scheduling and management of the activities (Kraeling and Webel, 2015) which contribute to the germplasm banks. Use only of altrenogest in gilts showed results of a lower estrus interval (2-3 d) and higher ovulation rate (Wang et al., 2018).

The use of eCG, in this study, served the propose to induce superovulation, as the animals that received eCG showed a higher number of ovulations, compared to the other treatment groups, and since Brazilian Piau breed has average of 11.1±2.4 in ovulation rate, number of total pigs born (9.3±2.7) and pigs born alive (7.9±2.6) (Silva et al., 2011). Also seen in other studies with averages of more than 20 ovulations in commercial breeds, when the dosage applied was equal to or greater than 1000 IU (Besenfelder et al., 1997; Hazeleger et al., 2000; Rátky et al., 2001; Fujino et al., 2006; Kauffold et al., 2007; Sommer et al., 2007; Martinat-Botté et al., 2010). Given that eCG activity is similar to FSH and LH (Brüssow et al., 2009; Soede et al., 2011; Driancourt et al., 2013; De Rensis and Kirkwood, 2016), a dosage of 1000 IU triggers greater follicular growth, stimulating greater numbers of follicles to grow and develop LH receptors and, subsequently, to ovulate, resulting in a larger amount of CL.

On the other hand, animals that received eCG had a great amount of anovulatory follicles. The molecular mechanism for the formation of follicular cysts is not yet known (Ziecik et al., 2017). Studies with sheep show that a high dose of eCG may cause anovulatory follicle development, probably due to the long half-life of this gonadotrophin (Jabbour and Evans, 1991). However, studies with doses of 1000 IU in swine have been effective for superovulating the animals without the occurrence of anovulatory follicles (Hazeleger et al., 2000; Rátky et al., 2001; Fujino et al., 2006). These results differ from those found in this study, wherein those animals which received 1000 IU of eCG (eGC+GnRH group) showed a greater number of anovulatory follicles than those which did not (P4 and GnRH groups). The use of a locally adapted breed, rather than a commercial breed as in other studies, may be largely responsible for this divergence of results, since the local breeds are smaller and therefore have a lower metabolic demand than commercial breeds, making an eCG dose of 1000 IU possibly excessive.

Given that the breeds studied have fewer ovulations than commercial breeds, this study sought to stimulate follicular development via the use of eCG in order to increase embryo production. However, the proportion of viable embryos in the group of animals in which follicular development was stimulated with eCG was significantly lower than in the groups receiving progestins and GnRH, which is similar to the results of another study (Wollenberg et al., 1990). These results suggest that the small proportion of viable embryos and, consequently, of freezable embryos in eCG+GnRH group may be due to higher plasma concentrations of E2 due to the large number of anovulatory follicles, which could produce a change in the uterine tubes and the uterus which would adversely impact fertilization and embryo quality.

In addition, the recovery rate of the animals in the eCG+GnRH group was lower than in other studies (Herrmann and Holtz, 1981; Schlieper and Holtz, 1986; Wollenberg et al., 1990; Hazeleger et al., 2000; Fujino et al., 2006; Kirkwood and Kauffold, 2015; Hribal et al., 2016) and lower than that of the other groups in this experiment. Some hypothesis could be made, due to the larger amount of CL and anovulatory follicles an imbalance may occur between E2 and P4 and it is known that these hormones influence the size and movement of the fimbriae, the contractility of the oviduct and the production of fluid (Barton et al., 2020). In some species as horses, rats and hamsters only embryos are transported to uterus, unfertilized eggs are retained in oviduct (Li and Winuthayanon, 2017). Failure to capture oocyte and embryo transport (He et al., 2019).

The use of 1000 IU of eCG stimulated follicular growth and hence superovulation. However, had a great amount of anovulatory follicles and the number of recovered structures reduced producing a lower response in quantity and quality of embryos showing a significant difference in the number of total structures, viable and frozen embryos than the other groups. Therefore, it is probable that adjustments to the protocol and the hormone levels would improve embryo recovery and embryo quality.

## Conclusions

All protocols were effective for synchronizing estrus within four to seven days in cyclic gilts. Protocols that used progesterone and GnRH were effective in the production and recovery of embryos. Furthermore, the protocol including the use of GnRH can also enable the use of artificial insemination and embryo collection at a fixed time.
